# The effect of nisin on the biofilm production, antimicrobial susceptibility and biofilm formation of *Staphylococcus aureus* and *Pseudomonas aeruginosa*

**DOI:** 10.1186/s40001-022-00804-x

**Published:** 2022-09-08

**Authors:** Parnia Ghapanvari, Mohammad Taheri, Farid Aziz Jalilian, Sanaz Dehbashi, Aram Asareh Zadegan Dezfuli, Mohammad Reza Arabestani

**Affiliations:** 1 Microbiology department, Faculty of Medicine, Hamadan University of Medical Sciences, Pajoohesh junction, Hamadan, Iran; 2 Department of Virology, School of Medicine, University of Hamadan, Pajoohesh junction, Hamadan, Iran; 3 Department of Microbiology, Faculty of Medicine, Ahvaz Jundishapour University of Medical Sciences, Ahvaz, Iran; 4 School of Medicine, Nutrition Health Research Center, Hamadan University of Medical Sciences, Hamadan, Iran

**Keywords:** Bacteriocin, Nisin, *Staphylococcus aureus*, *Pseudomonas aeruginosa*, Co-culture, Polymicrobial infection

## Abstract

**Objectives:**

*Staphylococcus aureus* and *Pseudomonas aeruginosa* were the most common bacteria in nosocomial infections. Different bacteriocins are currently being studied as antibiotics or in conjunction with antibiotics as potential strategies to treat resistant infectious agents. The study aimed to determine nisin's effect on the biofilm production, antimicrobial susceptibility, and biofilm formation of *S. aureus* and *P. aeruginosa*.

**Materials and methods:**

The experimental research tested two antibiotic-resistant isolates of *S. aureus* and *P. aeruginosa* strains. The experimental study tested two antibiotic-resistant isolates of *S. aureus* and *P. aeruginosa* strains. The MIC of bacteriocin nisin was determined using the micro broth dilution method, and crystal violet was used to assess the effect of bacteriocin on the biofilm. In addition, L929 cell culture was used to determine the effectiveness of bacteriocin on the isolate under similar cell conditions. Moreover, the MTT assay was used to and evaluate bacteriocin toxicity. In this study, the software Prism version 9 and Graph pad software were utilized.

**Results:**

The results of this study reveal that the nisin has different activities at different doses and is considered dose-dependent. At various times and doses, nisin inhibits biofilm formation in *S. aureus*, and *P. aeruginosa* isolates. Nisin also showed a decreasing survival of the isolates. Antibiotic-resistant bacteria can be made more vulnerable by nisin. Furthermore, nisin treatment affected the production of virulence factors such as hemolysins in *S. aureus* and had little or a negative effect on *P. aeruginosa* virulence factors. This medication stops *S. aureus* and *P. aeruginosa* from growing and causes bacterial cell damage.

**Conclusions:**

Antibacterial properties of nicin against *S. aureus* and *P. aeruginosa* were successfully studied. This bacteriocin stops *S. aureus* and *P. aeruginosa* from growing and causes bacterial cell damage or death. Damage to the membrane among the fundamental causes is reduced membrane potential and enzyme inactivation.

**Supplementary Information:**

The online version contains supplementary material available at 10.1186/s40001-022-00804-x.

## Introduction

Bacteriocins are antibacterial peptides formed by the ribosome of bacteria. They are a part of their pathogen-fighting immune system. Bacteriocin acts on preformed biofilms and may prevent them from forming [1]. Bacteriocin affects the bacterial cell surface and produces a pore, as well as the cell's permeability and bacteriostatic function [2]. Bacteriocins are classified into three groups, with a fourth group consisting of massive complexes containing carbohydrate or lipid components [3, 4]. The first class of bacteriocins is proteins that are made up of 19 to 50 amino acids and are significantly post-translationally modified [5]. Class, I bacteriocins called lantibiotics [2]. Nisin is a most popular class I bacteriocin [6]. Nisin can be produced by gram-positive bacteria such as *Lactococcus lactis* and some *Streptococcus* spp. [1]. The mechanism of action of nisin is the formation and fatal loss of membrane potential. Nisin is used as a food preservative in processed foods and has been listed in the generally recognized as a safe category by the US Food and Drug Administration (FDA).

Furthermore, it can be used with antibiotics to treat patients infected with antibiotic-resistant bacteria such as methicillin-resistant *Staphylococcus aureus* and as an agent to control biofilm formation by *S. aureus* and other pathogens [1]. Other studies also show that bacteriocin nisin has a more significant effect on microorganisms such as *M. luteus* and *L. lactis*, 17 and also against *B. subtilis, S. aureus*, MRSA, and VRE18 than hybrids or linear peptides [7] and has the power to inhibit the expansion of Gram-positive bacteria, comparable to *Listeria monocytogenes*, *S. aureus*, and *Streptococcus* spp. [8]. Many scientists, have studied the effect of the bacteriocin nisin on MRSA strains. They concluded that nisin alone or in combination with conventional antibiotics, such as vancomycin or ciprofloxacin, is considered a good candidate for further research [9, 10].

In humans, *S. aureus* is a commensal bacterium found in the nasal cavity, skin, and intestine. It is also capable of causing opportunistic infections, such as suppurative disease, pneumonia, and sepsis [3]. Over the last decade, methicillin-resistant *S. aureus* (MRSA) and community-acquired MRSA (CA-MRSA) have become major public health concerns. In addition, a common source of bacterial infections, causing anything from minor skin infections to life-threatening invasive diseases [1].

The relationship between *S. aureus* and *P. aeruginosa* has been extensively studied using infection models [3]*. P. aeruginosa* is an opportunistic pathogen predominantly associated with nosocomial infections [4]. *P. aeruginosa*, such as *S. aureus*, increased inflammation in kids [2]. In addition, early *P. aeruginosa* colonization is linked to increased exacerbation and morbidity [3, 4]. Early stage colonization can be aided by proteases, rhamnolipids, phospholipase C, hemolysin, and other virulence factors in *P. aeruginosa*. During chronic infection, the expression of these virulence factors declines over time, allowing the bacteria to persist [5]. Early in life, *P. aeruginosa* acquisition is associated with increased isolation of methicillin-resistant *S. aureus* [1]. Usually, the prevalence of *S. aureus* in CF patients drops sharply during late adolescence and adulthood, with *P. aeruginosa* emerging [2]. This striking negative clinical correlation between *S. aureus* and *P. aeruginosa* has driven several in vitro and in vivo studies geared toward characterizing the interbacterial interactions of these two organisms [3–6, 11–13]. According to a previous study, *P. aeruginosa* produces antistaphylococcal products and proteases, such as LasA, that can cause *S. aureus* biofilm dispersion and cell lysis [3–5]. *P. aeruginosa* also contains hydrogen cyanide, quinoline N-oxides, and phenazine pyocyanin, inhibiting *S. aureus* respiration [6, 11, 12]. Despite intensive antibiotic treatments, *P. aeruginosa* infections are challenging to eradicate. The antibiotic treatment may favor the emergence of antimicrobial drug resistance [13].

To date, studies of these two organisms have characterized isolated interactions using various in vitro or in vivo models. Therefore, the present study aimed at the effect of nisin on the biofilm production, antimicrobial susceptibility, and biofilm formation of *S. aureus* and *P. aeruginosa*.

## Main text

### Materials and methods

#### Strains and cultural conditions

Bacteriocin Nisin produced by *L. lactis* strains was purchased from Sigma Aldrich. We isolated a strain from the resistance species of *P. aeruginosa* and *S. aureus* stored in the Hamadan University of Medical Sciences microbial bank. This isolation was based on the virulence factor profile (biofilm formation and toxin production) and the antibiotic resistance pattern of mentioned bacteria. *S. aureus* ATCC25923 and *P. aeruginosa* PAO1 were used as the control strains. Trypticase soy broth (TSB), mannitol salt agar (MSA), and cetrimide agar (CA) (Merck, Germany) were used to the cultivation and recovery of S*. aureus* and *P. aeruginosa* isolates. The cultured media were incubated with ambient air at 37 °C.

#### Minimum inhibitory concentration

Minimum inhibitory concentration (MIC) was carried out in 96 well microtiter plates in triplicate. Briefly, bacteria isolates were grown overnight in the appropriate conditions and medium. The fresh broth media and bacteria with OD_600_ of ∼0.5 were added to every well to attaining a final concentration of 10^5^ CFU ml − 1 in a volume of 0.2 ml. Nisin was dissolved in sterile distilled water to prepare stock with a 512 µg/ml concentration. This stock was used to provide dilutions 256–0.025 µg/ml by serial dilution. Then, target strains were cultured for 16 h at 37 °C. In addition, culture conditions were applied for control [6]. The MIC of nisin was evaluated for mono and co-culture of isolates.

#### Nisin effect on biofilm and death—growth curve experiments

For growth experiments, the overnight bacterial cultures were resuspended and adjusted to OD600: 0.1.50 μl in 1 ml TSB of each *S. aureus* and *P. aeruginosa*. These bacterial cultures were then placed into 96-well microtiter plates containing the appropriate nisin concentrations. Mono-cultures, and co-cultures were used as growing methods for both the clinical and control strains. After that, the microtitre plates were grown for two periods at 37 °C (24 h—48 h for death growth and 24–144 h for biofilm experiments). After incubation, the planktonic bacteria were sampled and diluted by phosphate-buffered saline (PBS). These dilutions were cultured on MSA and CA to measure the viable cell count of *S. aureus* and *P. aeruginosa* strains. Fresh PBS (200 l) was added to the plates, the cells were scraped with a scraper to disturb the biofilm, and all protocols for a viable cell count of planktonic bacteria were repeated. Growth curves were initially used to ensure that all isolates could be survived and be developed in the TSB medium, planktonic, and biofilm phase [11].

#### Planktonic and biofilm co-culture on the L929 cell line

A fibroblast cell line (L929) was employed to treat live cells, and the effects of nisin were examined on *S. aureus* and *P. aeruginosa*. L929 fibroblast cells were purchased from the Iran Pasteur Institute (Tehran, Iran). As well, DMEM medium (Gibco Invitrogen, USA) and 10% Fetal Bovine Serum (FBS) (Gibco Invitrogen, USA) were used for cell culture in atmospheric humidity and 5% CO2 (Incubation temperature: 37◦C). When cells reached 90% confluency in Cell Culture, The overnight culture of bacteria was washed in PBS and resuspended in 1 ml MEM with L-Glutamine. Then modified to an OD600: 0.1.200 μl of each *S. aureus* and *P. aeruginosa* strain for the cultivation methods of mono-cultures and co-cultures were applied for both of clinical and control strains. The relevant concentration of nisin was added. The microplate was incubated according to the instructions. The cells' culture medium was changed. The medium is replaced every 24 h with a new one. After the growth media and planktonic cells were aspirated, the biofilm cells were scraped from the plate's surface with a cell scraper. Aspirated cells were diluted in fresh PBS and cultured on MSA and CA to recover the *S. aureus* and *P. aeruginosa* strains. This experiment was performed three times [2]. This test was performed before treatment with nisin to ensure bacterial growth and obtain pre-treatment information.

#### Biofilm

To measure the biofilm produced, the violet crystal method was used. As described in earlier sections, isolates were cultured in a single and simultaneous culture with the relevant concentration of nisin in 96 well microtiter plates. After 48 h of incubation, the supernatants were removed by washing the plates three times. All wells were filled with 100 l of 0.01% crystal violet solution. After 15 min of dyeing, the excess Crystal violet was removed by washing twice with sterile water. Eventually, the fixed Crystal violet was released by 95% ethanol. And then, the absorbance of the microtiter plate was measured by a spectrophotometer at 540 nm. The assay was repeated at least three times per strain [14].

#### Virulence factor production

In planktonic and biofilm states, pyocyanin, pyoverdine, biofilm, LasA protease, and hemolysin were investigated in the bacteria before and after treatment from the planktonic and biofilm conditions.

#### Pyocyanin

After culturing the samples in a convenient culture medium, the pyocyanin production rate was measured by the chloroform and HCl method, as explained by Mz el-foully [11]. To determine the Concentration of pyocyanin (µg/ml), The appendix formula was used = OD520 × 17.072.

#### Pyoverdine

The samples in RPMI 1640 medium (Invitrogen, USA) at 37 °C and 100 rpm were incubated overnight. The concentration of the culture medium was measured at OD 600 nm. Cultures were centrifuged at 200 g for 30 min. Subsequently, supernatants were filtered (Merck, Germany) (0.22 μm). The generation of pyoverdine was then measured at OD.405 nm. The following formula was used to measure to relative pyoverdine expression = OD. 405/OD. 600 [13].

#### Las B protease

The proteolytic activity of bacteria was determined by cultures on LB or BHI agar plates supplemented with 1% skim milk. After overnight incubation at 37 °C, The clear area around the colony was controlled the next day [15].

#### Hemolysin

Hemolysis production of *S. aureus* isolates was measured by spectrophotometry. In this method, 1 ml of washed red blood cell suspension was mixed with 500 μl of bacterial supernatant and incubated for 1 h at 37° C. The suspension was then centrifuged at 8000 RPM, and the absorbance of the supernatant at 560 nm was read [16].

#### MTT test

Skin fibroblast cell culture (L929) was used to evaluate the toxicity of nisin. After confluency of 90%, nearly 8,000 cells were added to each well. Then, nisin was added to the wells at 0.25–256 µg/mL concentrations. After 18 h of incubation at 37° C and 5% CO2, the culture medium was removed, then 50 μl of serum-free medium and 50 μl of MTT solution were added to the wells. After 3 h of incubation, 150 μl of MTT solvent was added. Fifteen hours after incubation in a shaker incubator, the light absorption of each well was read at OD590 [17]. The test steps are shown in a flowchart in supplementary files (F1, 2, 3).

#### Statistical analysis

The data were analyzed using GraphPad Prism software ver. 9 (graph Pad, USA). Two-way ANOVA, one-way ANOVA, and *t* students` tests were performed. Multiple comparison tests were done, where it was applicable. The data were presented as mean ± SEM.

### Results

#### Minimum Inhibitory concentration

The minimum inhibitory concentration (MIC) for nisin was determined regarding SA-1 and PA-1 in mono and co-culture. MIC is the lowest antibiotic concentration that visible inhibition growth of the target strain after 16 h at 37° C in the MHB. The MRSA isolate (SA-1) displayed high MICs to nisin (MIC = 256 µg/ml); in contrast, *S. aureus* ATCC 25,923 proved to be susceptible to the nisin (MIC = 2 µg/ml).

*P. aeruginosa* PAO1 displayed a MIC of 64 µg/ml. The multi-drug resistant *P. aeruginosa* (PA-1) was resistant to nisin. In co-culture SA-1/PA-1 exhibited MICs of 128 µg/ml (Table 1).

**Table 1:** Minimum inhibitory concentration (MIC) of nisin for isolates and strains

Strain	MIC(µg/ml)
SA-1	256 µg/ml
*S. aureus* ATCC 25,923	2 µg/ml
*P. aeruginosa* PAO1	64 µg/ml
PA-1	Resistance
SA-1/PA-1	128 µg/ml

#### Growth curve experiments and biofilm treatment with nisin

**Fig. 1:**
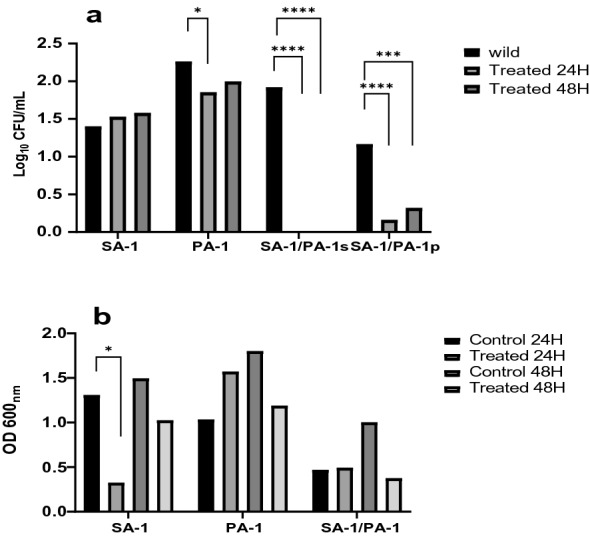
**a** Number of colonies of SA-1, PA-1, SA-1/PA-1 isolates in-vitro before and after treatment with nisin.SA-1: *S. aureus* isolate.PA-1: *P. aeruginosa* isolate. SA-1/PA-1 *S. aureus* isolated from co-culture. SA-1/PA-1 p: *P. aeruginosa* isolated from co-culture. **b** Distribution of biofilms of SA-1, PA-1, SA-1/PA-1 isolates before and after treatment with nisin

The viability of SA-1, PA-1, and SA-1/PA-1cells exposed to nisin for 24 and 48 h was determined using time-kill assays. For all tested concentrations of nisin (Less and more concentration MIC), the viable count for SA-1 increased during the 24 and 48 h (Fig. 1a). The viable cell count, in particular, increased significantly. During the first 24 h of time, PA-1 showed a reduction in the viable count. The total of viable cells was reduced significantly,; the next, in co-culture SA-1/PA-1, none grew after nisin treatment. Lack of growth may be due to the effect of nisin as well as the co-culture of *S. aureus* and *P. aeruginosa* (Fig. 1a).

For analysis of the effect of bacteriocin nisin on biofilm formation and destruction, biofilm degradation was measured. Biofilm formation of PA-1 and SA-1 in monoculture and co-culture is shown in Fig. 1b. The results showed that nisin destroys the biofilm of SA-1 isolated after 24 and 48 h, while only 48 h after incubation, it can destroy the biofilm of PA-1 isolate. SA-1 biofilm was destroyed after 24 h, but over time it seemed that SA-1 bacteria had adapted to the conditions, so that after 48 h, the biofilm destruction rate decreased. PA-1 biofilm production increased 24 h after treatment. In co-culture, biofilm degradation occurred after 48 h. The effect of nisin seems to be time-dependent, and the rate of biofilm degradation increases over time. In addition, as shown in Fig. 1b, the rate of biofilm production in simultaneous culture is less than single culture.

**Fig. 2:**
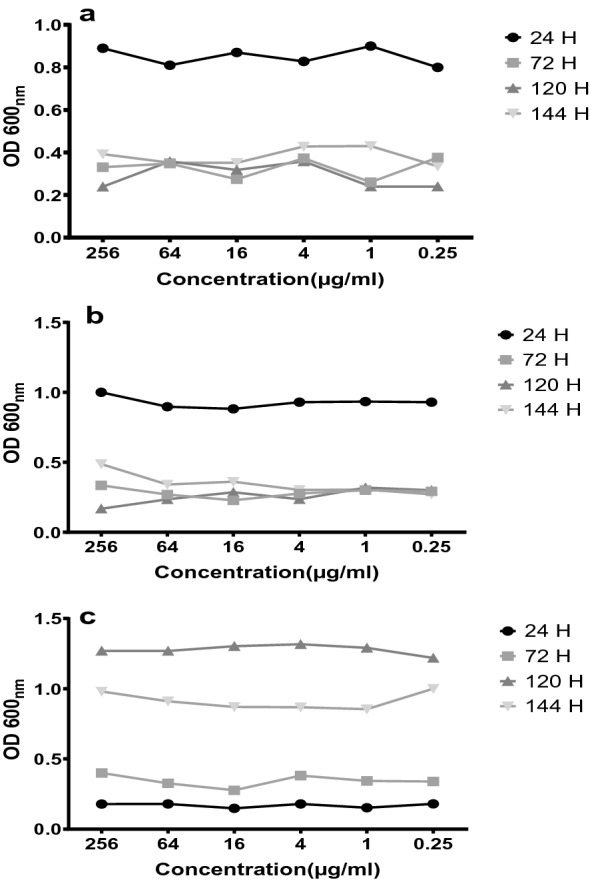
Inhibition of biofilm production at various concentrations of nisin In the period 24–144 h for *S. aureus *(**a**), *P. aeroginosa* (**b**) and co-culture *S. aureus* and *P. aeroginosa* (**c**)

In addition, greater biomass was evaluated for untreated cells than treated cells. The data indicated that nisin could efficiently prevent SA-1 and PA-1 biofilm formation in mono and co-culture and disrupt established biofilm at its MIC value (Fig. 2).

#### Growth curve experiments treatment with nisin on the L929 cell line

**Fig 3:**
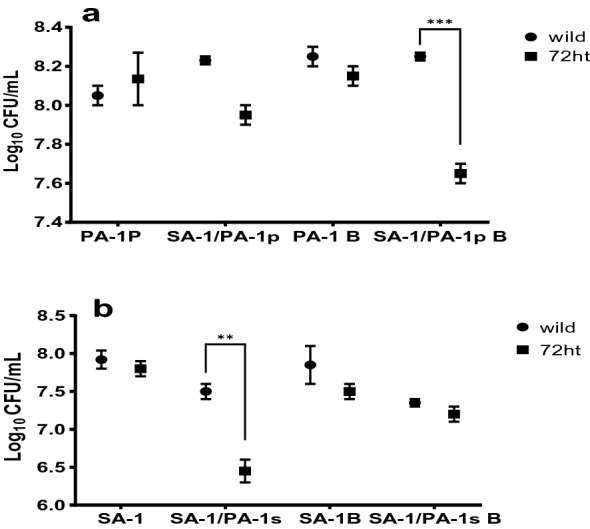
**a** Number of colonies of PA-1, SA-1/PA-1p isolates in cell culture, before and after treatment with nisin.PA-1P: *P. aeruginosa* Planktonic. SA-1/PA-1 P: *P. aeruginosa* Planktonic isolated from co-culture. PA-1 B: *P. aeruginosa* biofilm. SA-1/PA-1p B: *P. aeruginosa* biofilm isolated from co-culture 3b: number of colonies of SA-1, SA-1/PA-1 s isolates in cell culture, before and after treatment with nisin. SA-1P: *S. aureus* Planktonic. SA-1/PA-1P s: *S. aureus* planktonic isolated from co-culture. SA-1 B: *S. aureus* biofilm. SA-1/PA-1 s B: *S. aureus* biofilm isolated from co-culture.

According to the results, as shown in Fig. 3a, the rate of PA-1 biofilm in co-culture of SA-1/PA-1 decreased after 72 h of treatment, demonstrating nisin's effect on PA-1. In addition, as shown in Fig. 3b, in the co-culture of SA-1/PA-1, the number of SA-1 isolated planktonic after 72 h of treatment with nisin decreased significantly, and this diversity is significant. These results showed an inhibitory effect exerted by the nisin and the PA-1 strain on the SA-1 strain.

#### The effect of nisin on virulence factors during co-culture

##### Hemolysin

**Fig. 4:**
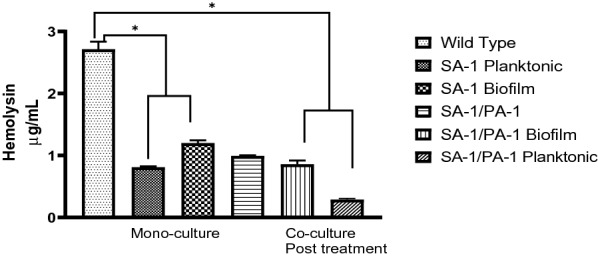
Produced hemolysin by SA-1 and SA-1/PA-1 s before and after treatment with nisin. Wild type: non-treated *S. aureus*. SA-1 planktonic: treated *S. aureus* planktonic. SA-1 biofilm: treated *S. aureus* biofilm.SA-1/PA-1: non-treated *S. aureus* isolated from co-culture. SA-1/PA-1 biofilm: treated *S. aureus* biofilm isolated from co-culture. SA-1/PA-1 planktonic: treated *S. aureus* planktonic isolated from co-culture

In this test, the effect of nisin on hemolysin of SA-1 before and after treatment was investigated. In Fig. 4, the results showed that hemolysin of SA-1 decreased after treatment, and a statistically significant discrepancy was observed.

##### Pyoverdine

**Fig. 5:**
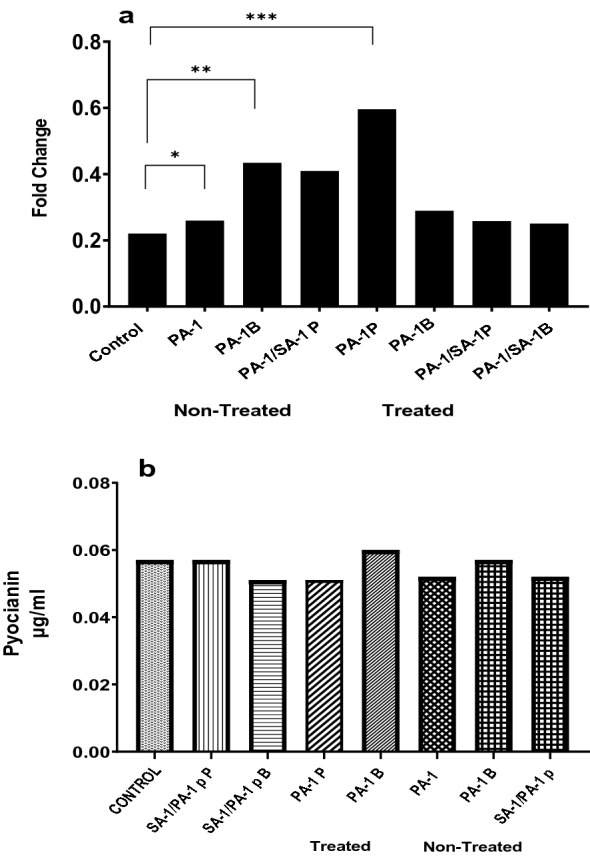
**a** Pyoverdine output before and after treatment with nisin. PA-1: *P. aeruginosa* non Treated. PA-1B: *P. aeruginosa* biofilm. SA-1/PA-1pP: *P. aeruginosa* Planktonic Treated isolated from co-culture. SA-1/PA-1pB: *P. aeruginosa* biofilm treated isolated from co-culture. **b** Pyocyanin output before and after treatment with nisin. SA-1/PA-1pPt: *P. aeruginosa* planktonic treated isolated from co-culture. SA-1/PA-1pBt: *P. aeruginosa* biofilm treated isolated from co-culture. PA-1Pt: *P. aeruginosa* planktonic treated. PA-1Bt: *P. aeruginosa* biofilm treated.

The results in Fig. 5a showed that the production of Pyoverdine in PA-1 isolates treated with nisin in the planktonic state has an enhancement, statistically considerable, and was ineffective.

##### Pyocyanin

Pyocyanin was appraised as a virulence factor produced by PA-1 before and after treatment with nisin. As shown in Fig. 5b, nisin did not affect this virulence factor, and no main discrepancy was observed.

#### Protease Las B

The results showed that Las B production was positive in all treated and untreated, and also, treatment with nisin did not affect Las B production.

#### MTT test

**Fig. 6:**
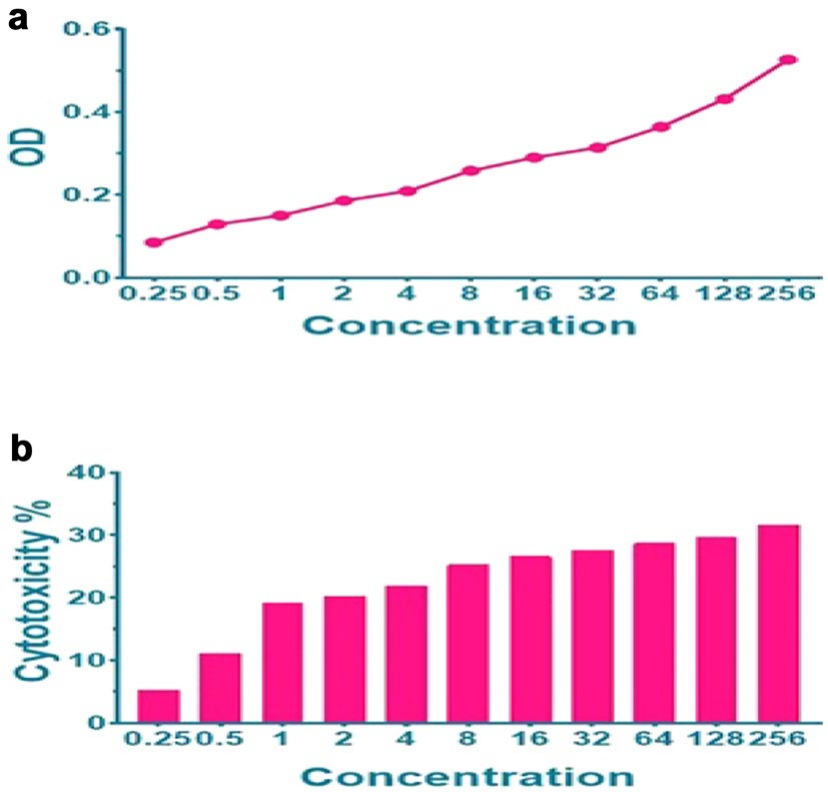
MTT test results show dose-dependent toxicity. Therefore, by increasing the dose of nisin, toxicity increases. As the data show, increasing the concentration of nisin increases the toxicity and IC50 nisin is more than 256 µ/ml

The MTT toxicity was dose-dependent, as can be seen in Fig. 6. Therefore, by increasing the dose of nisin, toxicity was increased, but the concentrations used in this experiment were less than IC50 and can be used up to 256 µ/ml in cell line L929.

### Discussion

The most prevalent bacteria isolated from chronic wounds are *S. aureus* and *P. aeruginosa*. They can produce virulence factors and surface proteins that slow the healing process. *S. aureus* and *P. aeruginosa* co-infection are more dangerous than a single infection. In addition, bacteria have innate and acquired antibiotic resistance, making infection hard to remedy, especially in patients with underlying diseases [18]. One of the most important virulence factors is biofilm. Biofilms are polymicrobial populations adherent to a surface and contain themselves in hydrated extracellular polymeric materials[19]. And make treatment difficult and sometimes impossible. Using bacteriocin, such as nisin and the lantibiotic, is considered one way to treat, complex, and impossible infections. It has antibacterial activity across a wide range of bacteria, including methicillin-resistant *S. aureus* (MRSA) and vancomycin-resistant enterococci (VRE)[7].

#### MIC

Contrary to the results of our study, the finding of research that was studied by Sibel Dosler et. al. and Regina Geitani et. al. reported the MIC of nisin for the isolate of *S. aureus* was 4 to 16 mg/l and > 128 µg/ml, respectively [9, 20]. This amount rate was comparatively lower than the reports of Karim Naghmouchi et al. [21]. In our study, the lowest MIC of nisin observed was for *P. aeruginosa* ATCC 25,923 (2 µg/ml), while the highest one observed was for SA-1 isolate (256 µg/ml). In addition, the PA-1 isolate was resistant to nisin. Moreover, MIC for co-culture SA-1/PA-1 Was (128 µg/ml). One of the most important reasons for this discrepancy was the type and quality of nisin used and mutations in bacteria isolates. It is important to point that the isolates which studied in this discussion were taken from a wound sample.

#### Effect of nisin on viability and biofilm of *S. aureus* and *P. aeruginosa*

We have investigated the in-vitro activity of nisin against SA-1, PA-1, and SA-1/PA-1 at 24–48 h. As demonstrated in Fig. 1a, for 24 h, the number of colonies counted for SA-1 increased, and PA-1 decreased significantly (P < 0.05). SA-1/PA-1(co-culture) extremely decreased. It seems that PA-1 adapted to new conditions, and increased the number of colonies. Similar results were noted by other researchers, such as Serena A. Mitchell et al. stated that nisin was more effective than any of the other linear hybrids or peptides but still showed less activity on *S. aureus*, MRSA, and VRE18 [7].

Contrary to our results, M. L. CABO et al. explained that nisin did not significantly affect *P. aeruginosa* [22]. The discrepancy in the data could be due to the geographical differences, the type of the investigated isolates, and their gene expression patterns. It is also crucial to utilize high-quality nisin. In addition, under the influence of co-culture, the development of SA-1 and PA-1 produces a variety of consequences.

In the present study, the results obtained that the rate of biofilm formation in the simultaneous culture was much less than the monoculture of any isolate. In addition, nisin after 24 h causes the destruction of the biofilm of SA-1 in the monoculture. After 48 h, destruction of PA-1 biofilm in monoculture and co-cultured. Similar results were reported by other researchers such as Lalitha Biswas et al. [23] and Laura M. Filkins et al. [2] that showed the co-culture of *P. aeruginosa* suppresses aerobic metabolism and growth of *S. aureus* [23]. In addition, Pyocyanin, produced by *P. aeruginosa*, can cause death and reduce the number of *S. aureus*, thereby decreasing the biofilm produced in co-culture. In contrast to the findings of this investigation, Patrcia M. Alves et al. claimed that *S. aureus* biofilms in co-culture increased statistically considerably compared to monoculture; however, *P. aeruginosa* biofilm production did not rise significantly[24]. Due to the different abilities of several isolates to produce biofilms, our results showed the diverse results, this finding suggests that nisin has been potentially used to treat GNB, such as *P. aeruginosa*.

#### Kill time curve (KTC) in cell line

Consistent with our results, Stephanie DeLeon et al. found that the number of *S. aureus* colonies remains constant in a rich media such as a wound-like medium (WLM) compared to a culture medium, such as LB [25]. Also showed that SA-1 planktonic in co-culture on cell line compared with PA-1 better restrained by nisin.

#### Virulence factor production

Consequently, our findings showed that hemolysin production by SA-1 reduction after treatment with nisin, whereas Dieter Worlitzsch 's found that using antibiotics increased hemolysin production [26]. One of the reasons for the discrepancy in the outcome is that different antibiotics can change the transcription and translation patterns. These changes occur differently with nisin.

In addition, pyocyanin production as a virulence factor of PA-1 isolated before and after treatment with bacteriocin–nisin did not change. Pivordine production in PA-1 isolates treated with nisin in the planktonic state increased significantly (*P* < 0.05) and was statistically significant. Treatment with nisin did not affect Las B production. On the other hand, in Da-Hye Lee et al. study, the production of virulence factors by *P. aeruginosa* KCTC 2004 can suppress under the influence of bacteriocin[27].

The results of the MTT assay showed that increasing the dose of nisin also increases in the toxicity of nisin. Compared to the results of the present study, a study by Nam E. Joo et al. stated that nisin is not toxic to animals and is safe for human consumption. About 0.6 mg of nisin is safe for each person per day as part of a natural food intake [28]. Therefore, it can be concluded that the increase in toxicity of nisin in the present study is acceptable in therapeutic methods.

### Conclusions

Antibacterial properties of nisin against *S. aureus* and *P. aeruginosa* were successfully studied. This medication bacteriocin stops *S. aureus* and *P. aeruginosa* from growing and causes bacterial cell damage or death. Damage to the membrane among the fundamental causes are reduced membrane potential and enzyme inactivation. This chemical offers a lot of potential for usage in the food and medical industries as an antibacterial agent.

## Supplementary Information

**Additional file 1.** In flowchart, the tests steps are shown in a flowchart.

## Data Availability

All data generated or analyzed during this study are included in the present published article and its supplementary information file.

## Abbreviations

MRSA: Methicillin-resistant *S. aureus*
CA-MRSA: Community-acquired MRSA
TSB: Trypticase soy broth
MSA: Mannitol salt agar
CA: Cetrimide agar
MIC: Minimum inhibitory concentration
PBS: Phosphate-buffered saline
FBS: Fetal bovine serum
