# Supplementary material for: The effect of nisin on the biofilm production, antimicrobial susceptibility and biofilm formation of Staphylococcus aureus and Pseudomonas aeruginosa
Source: Eur J Med Res. 2022 Sep 8;27:173. doi: 10.1186/s40001-022-00804-x (PMC9461124; doi:10.1186/s40001-022-00804-x)
Supplement: Supplementary file 1 — Additional file 1. In flowchart, the tests steps are shown in a flowchart. [file 40001_2022_804_MOESM1_ESM.docx]

**Flowchart**:

**The tests steps are shown in a flowchart:**

Flowchart 1: Steps of control tests without treatment of strains

Flowchart 2: Steps of tests and treatment of strains with nisin in in-vitro condition.

Flowchart 3: Steps of tests and treatment of strains with nisin in Cell culture condition
